# Increasing Number of Cases Due to *Candida auris* in North Italy, July 2019–December 2022

**DOI:** 10.3390/jcm12051912

**Published:** 2023-02-28

**Authors:** Camilla Sticchi, Roberto Raso, Lorenza Ferrara, Elena Vecchi, Loredana Ferrero, Daniela Filippi, Giuseppe Finotto, Elena Frassinelli, Carlo Silvestre, Susanna Zozzoli, Simone Ambretti, Giuseppe Diegoli, Carlo Gagliotti, Maria Luisa Moro, Enrico Ricchizzi, Fabio Tumietto, Francesca Russo, Michele Tonon, Francesco Maraglino, Giovanni Rezza, Michela Sabbatucci

**Affiliations:** 1 A.Li.Sa. Azienda Ligure Sanitaria, Ligurian Health Authority, 16121 Genova, Italy; 2 Regional Epidemiology Reference Service for the Surveillance, Prevention and Control of Infectious Diseases—Local Health Unit of Alessandria, 15121 Alessandria, Italy; 3 Collective Prevention and Public Health Section—Directorate General for Personal Care, Health and Welfare—Emilia Romagna Region, 40100 Bologna, Italy; 4 S.C. Health Directorate—P.O. Molinette—A.O.U. Città della Salute e della Scienza, 10126 Turin, Italy; 5 Microbiology Unit, IRCCS Azienda Ospedaliero-Universitaria Bologna, 40138 Bologna, Italy; 6 Section of Microbiology, Department of Experimental, Diagnostic and Specialty Medicine, University of Bologna, 40126 Bologna, Italy; 7 Regional Health and Social Agency—Emilia Romagna Region, 40127 Bologna, Italy; 8 UO Antimicrobial Stewardship—AUSL Bologna, 40124 Bologna, Italy; 9 Veneto Region, Directorate for Prevention, Veterinary Food Safety, 30123 Venice, Italy; 10 Ministry of Health, Directorate General Health Prevention, Communicable Diseases and International Prophylaxis, 00144 Rome, Italy; 11 Department Infectious Diseases, Istituto Superiore di Sanità, 00161 Rome, Italy

**Keywords:** *Candida auris*, outbreak, epidemic, epidemiology, Italy, healthcare-associated infections

## Abstract

*Candida auris* is an emerging fungus that represents a serious health threat globally. In Italy, the first case was detected in July 2019. Then, one case was reported to the Ministry of Health (MoH) on January 2020. Nine months later, a huge number of cases were reported in northern Italy. Overall, 361 cases were detected in 17 healthcare facilities between July 2019 and December 2022 in the Liguria, Piedmont, Emilia-Romagna, and Veneto regions, including 146 (40.4%) deaths. The majority of cases (91.8%) were considered as colonised. Only one had a history of travel abroad. Microbiological data on seven isolates showed that all but one strain (85.7%) were resistant to fluconazole. All the environmental samples tested negative. Weekly screening of contacts was performed by the healthcare facilities. Infection prevention and control (IPC) measures were applied locally. The MoH nominated a National Reference Laboratory to characterise *C. auris* isolates and store the strains. In 2021, Italy posted two messages through the Epidemic Intelligence Information System (EPIS) to inform on the cases. On February 2022, a rapid risk assessment indicated a high risk for further spread within Italy, but a low risk of spread to other countries.

## 1. Introduction

The dissemination of *Candida auris* [1,2,3,4] and other multidrug-resistant organisms [5,6] is a growing public health threat worldwide, aggravated by the COVID-19 pandemic [7], that requires an improvement in microbiology laboratory capacity, integrated surveillance systems, and policy action.

*C. auris* has caused severe infections around the world. The earliest isolates of nosocomial infection were identified in 1996 in Korea [8] and in 2009 in Japan [9], while the identification of the first European case dated back to 2007 in France, and it was due to an isolate belonging to the South Indian clade I [10]. 

Since then, the reporting of cases was increasing rapidly worldwide. In the European Economic Area (EU/EEA), 620 cases were reported in the period January 2013–December 2017 [11], and 349 cases were reported between January 2018 and May 2019 [12]. In the period October 2021–September 2022, the United States reported 1994 clinical cases and 5071 screening cases [2], and its transmission is steadily increasing [13]. 

Worldwide, the following risk factors for *C. auris* infection have been described: male gender, prematurity, underlying diseases such as diabetes, kidney or ear disease, trauma, history of central venous catheters, and use of broad-spectrum antibiotics [14]. In the United States, *C. auris* has been predominantly identified among patients with extensive exposure to ventilator units by skilled nursing facilities and long-term acute care hospitals [15]. The clinical manifestation of *C. auris* infection depends upon the site of infection. It can cause wound infections and otitis. *C. auris* was found in urine and respiratory specimens, although its contribution to clinical disease in these sites is unclear, while bloodstream infection typically causes sepsis and severe illness. Invasive infections due to *C. auris* were associated with a 30–60% in-hospital crude mortality rate. In addition, *C. auris* can colonise patients’ skin and other body sites asymptomatically, and it is the only *Candida* species that is transmissible from patient to patient. Most of the *C. auris* strains were resistant to at least one out of the three major classes of antifungal drugs, one-third were resistant to two antifungal drug classes, and some strains were resistant to all of the three classes [16]. Particularly, antifungal-resistant *C. auris* strains increased by 60% in 2020 vs. 2019 in the US due to the COVID-19 epidemic. *C. auris* has caused numerous healthcare-associated outbreaks [17], also spreading among hospitalised patients with COVID-19 [18,19]. *C. auris* can persist in the healthcare environment for weeks, with reduced susceptibility against clinically relevant concentrations of chlorhexidine and hydrogen peroxide, and eradication is achieved using povidone iodine only [20,21].

The control of *C. auris* requires timely detection and adherence to recommended infection prevention and control (IPC) practices. Yeast identification methods used at many clinical laboratories often misidentify *C. auris* as other yeasts (e.g., *Candida haemulonii*) [22,23], thereby making its control challenging. A consensus case definition, which was approved in 2018 [24] and last updated in 2019 [25], allows for the standardised public health tracking and surveillance of *C. auris* cases, providing opportunity for rapid response to contain its spread. 

In Italy, cases of *Candida* spp., including *C. auris*, are subjected to mandatory notification according to the Ministerial Decree of 15 December 1990 “Information system for infectious and diffusive diseases” referring to infectious diseases included in the Class V. The first case of invasive *C. auris* infection was identified in 2019 [26]; then, other scientific articles described hospital outbreaks that occurred locally in recent years [27,28].

This report describes the demographic, geographic, and temporal variables associated with *C. auris* cases reported in Italy, together with the available microbiological results, with the aim to inform and raise awareness on this health threat and facilitate its prevention and control at the country and global level. 

## 2. Materials and Methods

Cases were reported by routine surveillance from the local healthcare facilities to the regional health authorities and/or the MoH through the regional electronic systems or email referring to the case definitions described by the European Centre for Disease Prevention and Control (ECDC) [17]. On October 2021, following the notification of cases from the region Liguria, the Italian MoH queried this region and the neighbours (Emilia-Romagna, Lombardy, Piedmont, and Tuscany) specifically for retrospective identification and notification of cases. Passive notification of cases from all the interested Regions through the country continued. Here, we carried out a national descriptive study of cases infected or colonised by *C. auris* identified in Italy between July 2019 and December 2022.

Confirmed cases were individuals with *C. auris* identified by Matrix-Assisted Laser Desorption Ionisation Time of Flight (MALDI-TOF Bruker), VITEK 2 YST (bioMérieux), or Polymerase Chain Reaction (PCR) performed in clinical samples or surveillance cultures according to the referred diagnostic methods [3,22]. Contact tracing was performed for patients admitted in hospital wards where a confirmed case was hospitalised. All the cases, with the exception of the only one imported, had epidemiological (being hospitalised in the same ward/hospital regardless of temporal link).

Each involved region sent to the MoH the demographic, microbiological, and clinical data by one excel file. Data were anonymised at the local level. We performed a descriptive analysis by the R statistical software (ver. 4.1.1). The ECDC Map Maker tool (EMMa) was used to create the map of the areas with resident cases. The number (N) of the subjects with data available was detailed in Table 1 for each demographic and clinical characteristic.

Antifungal susceptibility testing was performed by using MICRONAUT-AM broth microdilution panels (MERLIN Diagnostika GmbH, Bornheim, Germany). According to the European Committee on Antimicrobial Susceptibility Testing (EUCAST) clinical breakpoints [29], we defined antifungal resistance as referred to Fuconazole, while non-species related breakpoints have been determined on the basis of PK/PD.

All the high-touch surfaces in the patient area were sampled and tested regularly after the notification of each colonised/infected case.

Incidence rates were calculated by dividing the number of cases by the number of the resident population per region. The year 2021 was selected as the mid-year in the referred period mid 2019–end of 2022. Regional demographic data were obtained by the Italian National Institute of Statistics (ISTAT, https://demo.istat.it/index_e.php, accessed on 1 December 2022). The resident population included both Italian and foreign individuals resident in Italy even if temporarily absent. 

## 3. Results

### 3.1. Notification of Cases

The first confirmed case due to *C. auris* occurred in a hospital of the Liguria region in 2019 [26] and was reported to the MoH on January 2020. A literature search in PubMed revealed some cases occurred in the same hospital in 2020 [30,31], while the Region Liguria where the hospital was located reported cases on February, September, and October 2021. Then, following the Ministerial request of retrospective investigation, Liguria reported a huge outbreak with 277 cases infected or colonised by *C. auris* occurring between November 2020 and October 2021 [32]. Moreover, 83 more cases were reported in the period November 2021–December 2022 (19 by Liguria, 48 by Piedmont, 15 by Emilia-Romagna, and 1 by Veneto) for a total of 361 cases. 

The last *C. auris* cases were reported on December 2021 in Emilia-Romagna, on June 2022 in Veneto, on September 2022 in Liguria and on December 2022 in Piedmont.

### 3.2. Characteristics of Cases Colonised or Infected by C. auris

The index case identified in Liguria was reported by Crea et al. in 2019 [26]. The patient had no history of recent travel abroad or hospital admission. 

Table 1 describes the main demographic and clinical characteristics of confirmed *C. auris* cases reported in Liguria, Piedmont, and Emilia-Romagna regions, while only one case was reported by the Veneto region in June 2022. The number of the subjects (N) for whom data were available was detailed for each variable and considered as a denominator for the proportion of the specific variable listed. Overall, the mean age of the cases was 61.8 years (median age 64 years, age range: 0–91, N = 358), and most of them (66.3%, N = 237) were males. All the cases were hospitalised; most of them (79.1%, N = 284) were admitted to an Intensive Care Unit (ICU). One-third of the cases had no symptoms related to *C. auris* (33.9%, N = 116), had COVID-19 or tested SARS-CoV-2 positive (30.1%, N = 103), and some of them suffered from neurologic disorders (19.0%, N = 65), underwent surgery (8.4%, N = 30), and had sepsis/septic shock (9.4%, N = 32), cardiac (7.6%, N = 26) or acute respiratory disease (7.6%, N = 26).

Particularly, the variations in time elapsed between the date of sampling and the date of diagnosis follow: Liguria, median time 2.0 days (mean 2.8 days, range 0–7 days, calculated for N = 26 cases); Piedmont, median time 0 days (mean 0.1 days, range 0–7 days, N = 48); Emilia-Romagna, median time 2.5 days (mean 2.8 days, range 2–5 days, calculated for N = 12 cases).

Patients who died with/by *C. auris* were mid-age adults (Liguria region: mean age 64.9 years old, median age 70.6, age range 0–84, data available for N = 28 cases; Piedmont region: mean age 64.6 years old, median age 63, age range 43–82, N = 20; Emilia-Romagna region: mean age 61.7 years old, median age 59.5, age range 47–87, N = 6). These patients were hospitalised in the following wards: in Liguria, almost two-thirds of the patients (65.5%) in ICU, 4 (13.8%) Surgery, 4 (13.8%) Internal Medicine, 2 (6.9%) Emergency Department; in Piedmont, they were all admitted to ICU; in Emilia-Romagna, 5 were hospitalised in ICU and 1 was in Geriatrics.

**Figure 1:**
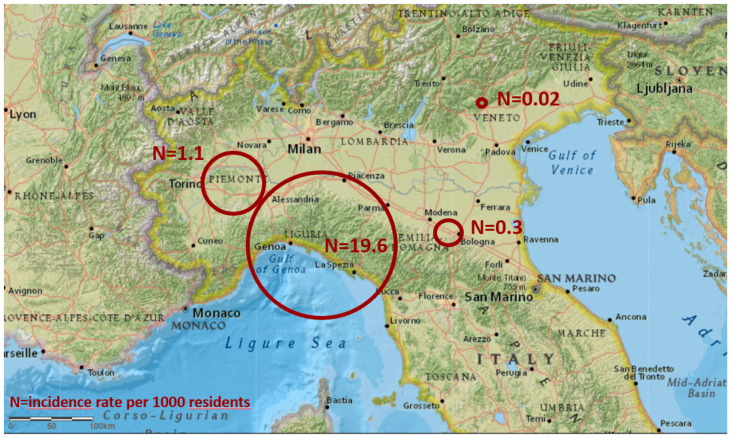
Map of the areas from where confirmed cases colonised with or infected by *C. auris* were reported, located in 4 neighbouring regions, North Italy, July 2019–December 2022 (N = 361). The size of the red circles represents the regional incidence rate per 1000 residents (N).

Overall, 361 cases were reported by 17 healthcare facilities located in 4 regions (297 cases distributed in 13 facilities in Liguria, 48 cases in 1 hospital in Piedmont, 15 cases in 2 facilities in Emilia-Romagna, 1 case in 1 hospital in Veneto). Figure 1 describes the areas where the confirmed cases were reported, colonised with or infected by *C. auris*.

Overall, 82.3% of the cases were located in Liguria region, 13.3% in Piedmont, 4.2% in Emilia-Romagna and 0.3% in Veneto.

**Figure 2:**
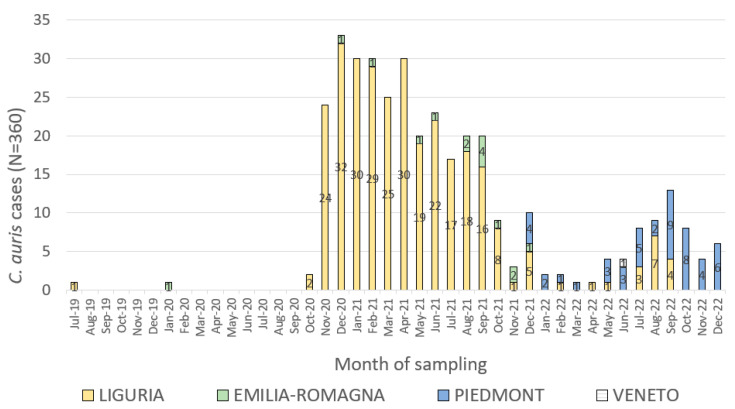
Epicurve of confirmed *C. auris* cases occurred in Liguria, Piedmont, Emilia-Romagna and Veneto regions by date of sampling and region of residence, Italy, July 2019–December 2022 (N = 360 out of 361 cases).

Figure 2 shows the epicurve of the cases occurred in the neighbouring four regions involved in the *C. auris* outbreaks in north Italy. Cases for whom the date of sampling was available are shown in the epicurve. 

The shape of the epicurve suggested an ongoing source epidemic that raised to a peak in December 2020, with a second lower peak occurred in September 2022. 

Table 2 reports the number of *C. auris* cases and the incidence rate per 1000 residents by region together with data on the number of residents and the number of beds in hospital facilities and nursing homes.

Table 2 describes the total number of cases and the size of the population resident in the four interested regions. 

### 3.3. Laboratory Testing

Among the microbiological methods reported for the diagnosis of *C. auris* infection/colonisation [3,22], MALDI-TOF was used mostly (126 out of 142 cases, 88.7%), while Biomerieux Vitek or PCR were used in very few cases (three out 79 reported, 3.8%, and two out of 79 reported, 2.5%, respectively). 

Antifungal susceptibility testing was performed on strains from infected patients rather than colonised individuals. All but one (85.7%, N = 7) isolate were resistant to at least one antifungal treatment (according to EUCAST breakpoints, fluconazole). Table 3 shows the microbiological results of the *C. auris* isolates tested against the main antifungal drugs. Data were available for seven strains from the Emilia-Romagna region, July 2019–December 2022.

### 3.4. Environmental Investigation

Environmental investigations were conducted in Emilia-Romagna and Liguria regions. All the environmental samples tested negative for *C. auris*. 

### 3.5. Control Measures

In each healthcare facility involved, the local infection prevention and control (IPC) teams put in place several health measures to control the spread of *C. auris*: standard and contact precautions, single room isolation or patient cohorting, dedicated staff and equipment where available, weekly screening of close contacts (sharing room, ward, non-disposable devices, and/or staff) until two weeks after the last case was discharged, internal audits, chlorine-based environmental cleaning and reprocessing of medical devices. The cleaning staff was instructed on the specific disinfection measures. Regular meetings of the health professionals were carried out as well as protocols for cases’ management were produced in the facilities involved. *C. auris* was included among the alert organisms. The patients and their families were informed on *C. auris* and provided with behaviour information at discharge.

In May 2018, following the Rapid Risk Assessment (RRA) by ECDC on *C. auris* infection, the Italian MoH published a circular letter to raise awareness on this health threat. The MoH informed the regions and autonomous provinces on the online platform of the ECMM (European Confederation of Medical Mycology) project FungiScope™ CandiReg [33], which enables international surveillance and facilitates the conduct of epidemiological studies, and nominated the National Reference Laboratory (Fondazione Policlinico Universitario A. Gemelli IRCCS Università Cattolica del Sacro Cuore) to support the characterisation of isolates and storage of strains. Then, the MoH organised meetings with the Italian regions involved and published five circular letters to give details for the notification of all the confirmed cases colonised/infected by *C. auris* isolated both in public and accredited healthcare facilities, microbiological information on this health threat, and epidemiological update while highlighting specific recommendations. 

To apprise the European countries, the MoH shared the first message through the web-based communication platform Epidemic Intelligence Information System (EPIS), which was later replaced by the European surveillance portal for infectious diseases (EpiPulse), on June 2021. A second update was posted on December 2021. In addition, dedicated meetings with the competent international health authorities (i.e., World Health Organisation—WHO, Transatlantic Taskforce on Antimicrobial Resistance—TATFAR, ECDC) were organised. 

Italy contributed to the third *C. auris* survey [34] conducted by ECDC on April 2022.

## 4. Discussion

Multiple transmission chains starting from different sources may have occurred simultaneously in the *C. auris* outbreaks described in Italy. Possibly, patients had common procedures in the same healthcare facility. The transfer of cases colonised with or infected by *C. auris* between facilities might have been unreported. Almost 1400 public or private healthcare facilities are located in Italy, of which over 10% are distributed in the two main regions with *C. auris* cases. Moreover, the capacity for the microbiological identification of *C. auris* and for the active surveillance of cases as well as the ability for patient isolation are highly heterogeneous through the country. However, Katja Saris et al. did not find any information of cases isolated in Italy in the period Jan 2016–July 2018 published in the scientific literature, including gray literature [35]. Some samples were processed by molecular typing, highlighting a recent introduction of *C. auris* in Italy and a rapid spread to some extent likely to be facilitated by the COVID-19 epidemic [30,31]. Particularly, around one-third of the cases overall had a positive SARS-CoV-2 test. Nevertheless, based on the regional trends of the epidemic, we observed that *C. auris* patients were diagnosed in periods when SARS-CoV-2 cases were less numerous than in others. We assumed that the decrease in *C. auris* cases between late-2021 and mid-2022 and then at the end of 2022 was attributed to several and repeated health interventions put in place in each involved facility. 

Given the high number of *C. auris* cases identified in Italy, the spread to several healthcare facilities and the difficulties to stop recurrence, on February 2022, the ECDC defined the risk of further spread within the country as high, while the risk of spread into other European areas was defined as low, unless there was a cross-border transfer of hospitalised patients. However, the epidemiological situation is rapidly deteriorating at the global level [34,36,37,38], and continued vigilance is needed. *C. auris* is an urgent health threat due to its global and rapid emergence, challenging microbiological identification, high mortality, and persistent transmissions. In the ECDC point prevalence survey on healthcare-associated infections in European acute care hospitals performed in 2011–2012, *Candida* spp. was the fifth most common pathogen associated with septicaemias, which was isolated in 7.4% of all documented cases. Results from the ECDC surveys described over 1800 *C. auris* cases reported by 15 EU/EEA countries from 2013 to 2021. 

The COVID-19 pandemic likely intensified the spread of *C. auris* and hindered the detection of additional cases [39]. The increased spread in hospitals could be a result of staff and supply shortages as well as changes in IPC practices (e.g., re-use or extended use of gowns and gloves). Although there is evidence of limited recombination in nature among the five clades after their divergence [40], clinical isolates were shown to be highly resistant to antifungal treatments [41] and surface disinfection. In particular, the marine environment was recently identified as a natural niche for this pathogen, while stored apples treated with antifungal agents were pointed as a possible reservoir for the transmission of *C. auris*-resistant strains to humans [42]. Thus, effective measures to contain *C. auris* spread require a multi-disciplinary approach [43]. 

## 5. Conclusions

The epidemiological situation regarding *C. auris* in northern Italy is alarming. Large and prolonged outbreaks cannot be ruled out for the years 2021 and 2022. Outbreak investigations should be initiated to detect possible sources and transmission routes, to better describe the outbreak strain and to identify risk factors in hospitalised patients.

The spread of *C. auris* underlined the importance of several practices: prompt outbreak investigation, availability of dedicated staff and isolation rooms, correct microbiological identification [44,45], effective treatment [46], trained health personnel, rigorous application of IPC measures including hand hygiene and research for innovative disinfection procedures [47], accurate patient screening and retrospective surveillance, precise information of cases and their family as well as between healthcare facilities in case of patient transfer. Prompt reporting of cases to the competent health authorities may facilitate local and national alert and shared diagnostic protocols, with the aim of limiting further spread of multidrug-resistant pathogens nationally and beyond. *C. auris* cases and outbreaks occurred in several EU/EEA countries within a few years after the first cases were reported in the EU/EEA with evidence of inter-facility spread in two EU/EEA countries and an assessment of endemicity in at least one region in one country [11]. 

The rapid emergence of *C. auris* and the high likelihood for undetected cases warrant strengthened microbiological and epidemiological surveillance as well as coordinated preparedness.

## Figures and Tables

**Table 1:** Main demographic and clinical characteristics of confirmed *C. auris* cases, Liguria, Piedmont and Emilia-Romagna regions, Italy, July 2019–December 2022 (N = 360 out of 361 total cases).

Patients’ Characteristics (%)	Liguria N = 297	Piedmont N = 48	Emilia-Romagna N = 15	Total N = 360
Median age (years, range)	N = 295 64 (0–91)	N = 48 64 (22–82)	N = 15 61.5 (47–87)	N = 358 64 (0–91)
Gender	N = 297	N = 48	N = 12	N = 357
Males	195 (65.7)	32 (66.7)	10 (83.3)	237 (66.3)
Females	102 (34.3)	16 (33.3)	2 (16.7)	120 (33.6)
Sample type	N = 294	N = 48	N = 15	N = 357
Blood	25 (8.5)	2 (4.2)	3 (20)	30 (8.4)
BAL/bronchial aspirate	40 (13.6)	3 (6.3)	6 (40)	49 (13.7)
CVC	0	1 (2.1)	1 (6.7)	2 (0.6)
Sputum	1 (0.3)	0	0	1 (0.3)
Surgical wound	1 (0.3)	0	0	1 (0.3)
Swab (inguinal/axillary and/or rectal)	217 (73.8)	40 (83.3)	3 (20)	260 (72.8)
Urine	10 (3.4)	2 (4.2)	2 (13.3)	14 (3.9)
Hospital ward	N = 296	N = 48	N = 15	N = 359
Emergency room	6 (2)	0	0	6 (1.7)
Geriatrics	0	0	2 (13.3)	2 (0.6)
ICU	228 (77.0)	46 (95.8)	10 (66.7)	284 (79.1)
Internal medicine	31 (10.5)	1 (2.1)	0	32 (8.9)
Rehabilitation unit	2 (0.7)	0	3 (20)	5 (1.4)
Surgery	29 (9.8)	1 (2.1)	0	30 (8.4)
Main comorbidity/medical issue	N = 289	N = 48	N = 5	N = 342
No symptoms due to *C. auris*	54 (18.7)	48 (100)	14 (93.3)	116 (33.9)
Acute respiratory disease	19 (6.6)	5 (10.4)	2 (40.0)	26 (7.6)
Bacteraemia	9 (3.0)	2 (4.2)	2 (40.0)	13 (3.8)
Chronic respiratory disease	4 (1.4)	1 (2.1)	0	5 (1.5)
Cardiac disease	21 (7.3)	5 (10.4)	0	26 (7.6)
SARS-CoV-2 positive/COVID-19	89 (30.8)	14 (29.2)	0	103 (30.1)
Diabetes	3 (1.0)	2 (4.2)	0	5 (1.5)
Immunosuppressive disorder/Cancer	4 (1.4)	6 (12.5)	0	10 (2.9)
Intestinal/renal/mediastinal disorder	0	10 (20.8)	0	10 (2.9)
Neurologic disorder	62 (21.5)	2 (4.2)	1 (20.0)	65 (19.0)
Sepsis/septic shock	32 (11.1)	0	0	32 (9.4)
Surgery	38 (13.1)	0	0	38 (11.1)
Trauma	14 (4.8)	1 (2.1)	0	15 (4.4)
Median time between diagnosis and death (days)	6.0 *	10.0 ^§^	n.a.	n.a.
In-hospital lethality	119 (40.1)	20 (41.7)	6 (50.0)	N = 145 (40.3)

* the denominator is 29 deaths; § the denominator is 20 deaths; BAL broncho-aspirated lavage; ICU intensive care unit; CVC central venous catheter; n.a. data not available.

**Table 2:** Incidence rate of *C. auris* cases per 1000 residents and number of healthcare beds by region, Liguria, Piedmont, Emilia-Romagna and Veneto regions, Italy, July 2019–December 2022.

Region	N. of Cases	Incidence per 1000 Residents	N. of Residents (1 Jan 2021)	N. of Hospital/Healthcare Beds (1 Jan 2019)
Liguria	297	19.6	1,518,495	5723
Piedmont	48	1.1	4,274,945	16,513
Emilia-Romagna	15	0.3	4,438,937	17,308
Veneto	1	0.02	4,869,830	17,472
Total	361	0.024	15,102,207	57,016

**Table 3:** Minimum inhibitory concentration resulted from susceptibility testing of *C. auris* strains isolated in Emilia-Romagna region, Italy, July 2019–December 2022 (N = 7).

Antifungal Drugs	MIC Range (mcg/mL)
Triazoles	
Fluconazole	≤0.0019–8
Itraconazole	≤0.0312–1
Posaconazole	≤0.0078–0.25
Voriconazole (and other 2° generation azoles)	≤0.0078–2
Polyenes	
Amphotericin B	≤0.0312–2
Anidulafungin	≤0.0019–0.125
Caspofungin	0.0156–0.25
Micafungin	≤0.0019–0.12
Flucytosine	≤0.0625–0.5

MIC minimum inhibitory concentration.

## Data Availability

Data are unavailable due to privacy protection and ethical restrictions.
